# An Ethnic Variation in the Acceptance of Biological Disease-Modifying Therapies: A University Hospital Experience

**DOI:** 10.7759/cureus.15270

**Published:** 2021-05-27

**Authors:** Haresh Selvaskandan, Arumugam Moorthy

**Affiliations:** 1 Department of Cardiovascular Sciences, University of Leicester, Leicester, GBR; 2 Department of Nephrology, University Hospitals of Leicester NHS Trust, Leicester, GBR; 3 College of Life Sciences, University of Leicester, Leicester, GBR; 4 Department of Rheumatology, University Hospitals of Leicester NHS Trust, Leicester, GBR

**Keywords:** biologic treatment, biologic agents, biologic therapies, ethnic disaprities, auto-immune, ethnic

## Abstract

Ethnic variations in the outcomes of rheumatological diseases are well documented. While physiological differences may account for these disparities, attitude to treatment is also likely to be a significant modifiable contributor. We sought to determine if an ethnic variation exists in the uptake of biological disease-modifying anti-rheumatic drugs (DMARD) among a multi-ethnic cohort when offered in-person through a healthcare system free at the point of access.

We conducted a retrospective cross-sectional study of patients seen in a biologic therapy counselling clinic between December 2016 and April 2017. Clinic letters from consultations were reviewed, and data including ethnicity, language spoken, and decision to accept or reject the therapy were extracted. We chose to measure uptake over adherence, as we believe it is an earlier, more direct marker of attitudes to joint saving medications.

Ninety-one cases were included in the analysis. Over 13.2% (12/91) of the cohort declined a biologic treatment when it was offered as the standard of care for joint disease. Non-Caucasian patients accepted treatment less often than Caucasian (White British) patients (OR 0.265, CI 0.73-0.959, p = 0.043), as did those who did not speak English as a first language (OR 0.094, CI 0.18-0.497, p = 0.005). Age, sex, and diagnosis were well matched between those who accepted and declined therapy.

We demonstrate a disparity in the uptake of biologic therapies between the White British population and patients from other ethnicities. The reasons for this are likely multifactorial and could be related to socio-economic factors, language barriers, and cultural differences. Addressing this discrepancy is a crucial first step to tackling preventable disparities in the outcomes of rheumatological disease between different ethnicities.

## Introduction

Ethnic variations in the outcomes of rheumatological diseases are well documented [1,2]. As populations around the world become more diverse, this issue is becoming an increasingly critical one to address. While physiological differences may account for variations [3,4], attitude to treatment is also likely to be a significant modifiable contributor [2]. Indeed, patients from minority ethnic groups are less likely to receive conventional (non-biologic) disease-modifying drugs for rheumatological conditions, even after adjusting for confounders [1]. Understanding why this disparity exists is essential to bridging the gaps in health-related outcomes that can exist within multi-ethnic populations [5].

Ethnic variations in medication adherence have been extensively investigated [6-8]. However, few have explored ethnic disparities in the initial uptake of biologic disease-modifying anti-rheumatic drugs (DMARD; biologics). Initial uptake can be an objective alternative to adherence, which is often self-assessed or determined by proxy [9], for determining attitudes to joint saving medications. Furthermore, understanding and addressing factors that contributed to a patient's decision to decline treatment initially could ultimately improve adherence. We thus sought to determine if a variation in the uptake of biologics for rheumatological conditions existed between ethnicities, when the therapy was offered in person, within a system free at the point of access (National Health Service, United Kingdom). We explored this in a tertiary rheumatological centre in Leicester, an ethnically diverse city in the midlands of the United Kingdom, where addressing outcome disparities is crucial to equitable care.

This article was previously presented as a meeting abstract at the European Alliance of Associations for Rheumatology (EULAR) annual congress of rheumatology in 2017.

## Materials and methods

Study design and participants

This was a service evaluation designed as a retrospective, single-centre, cross-sectional study, performed at the University Hospitals of Leicester NHS Trust (UHL) Tertiary Rheumatology Centre. Data were collected retrospectively from all patients attending our specialist rheumatology biological therapy clinic, between December 2016 and April 2017. All patients who are deemed suitable for biological therapy by a consultant (attending) rheumatologist within UHL are referred to this clinic for further counselling and consent. These clinics are run by a team of experienced rheumatology specialist nurses and provide patients with more time within a consultation to have concerns or questions addressed. All patients seen in this clinic are adults (≥18 years) with a rheumatological disease of any aetiology. The team of specialist nurses remained unchanged throughout the study period. We did not limit this study to any one particular autoimmune disease to maximise our sample size and to facilitate the generalisability of our results across diseases. At the design stage, we acknowledged that understanding the precise reasons for any differences in acceptance of biologic therapy would require a qualitative study involving at least focus groups and semi-structured interviews. This study was therefore designed to be hypothesis-generating, to facilitate further qualitative work if needed.

The UHL Clinical Audit Department approved this project (identifier 8808e) and deemed no further consent was required given all data were collected and analysed anonymously and retrospectively. The principles of the declaration of Helsinki and the International Conference on Harmonisation Good Clinical Practice Guideline (ICH-GCP) were nevertheless followed. Subject data were de-identified upon collection and data anonymity was maintained throughout.

Outcomes of interest

The primary outcome of interest was the acceptance of a biologic agent following counselling in the clinic by a rheumatology specialist nurse. We further collected data on ethnicity, age, sex, diagnosis, and first language as variables with which our primary outcome could associate. 

Data collection

All data were collected from hospital clinical records (clinic letters and medical records). Medical records were requested to clarify any events that were uncertain from clinic letters. Patient identifiers were removed upon data collection.

Statistical analysis

Data were locked prior to analysis using IBM SPSS version 26 (IBM Corp., Armonk, NY). Categorical data are presented as frequencies with percentages. Ethnicity was classified according to the 17 categories listed in the 2001 United Kingdom Census and then summarised as White British and Other Ethnic Groups (OEG) for analysis. Univariate analyses assessing the association of therapy acceptance with ethnicity were completed through binomial regression tests, and correlation was determined using the Pearson correlation coefficient. Patients with missing data were omitted from the analysis of that variable.

## Results

Ninety-eight consecutive cases were identified, seven were excluded due to inadequate data availability leaving a total of 91 cases that were included in our analysis. The demographics of the cohort have been summarised in Table 1. Over 61.1% of the cohort was White British, with the remaining belonging to OEG. This broadly matched census data for Leicester City from 2011. Sex distribution was nearly equal (49% female). Rheumatoid arthritis (RA) was the most common diagnosis necessitating biologics (43.8%), followed by ankylosing spondylitis (30.3%), and psoriatic arthritis (19.1%).

**Table 1:** Summary of cohort demographics.

	Whole cohort	Therapy declined	Therapy accepted
Total, n (%)	91 (100)	12 (100)	79 (100)
Age (years)			
54 or lower	49 (53.8)	6 (50)	43 (54.4)
Sex			
Female	45 (49.4)	6 (50)	39 (49.3)
Diagnosis			
Ankylosing spondylitis	27 (30.3)	2 (18.2)	25 (32.5)
Psoriatic arthritis	17 (19.1)	2 (18.2)	15 (19.5)
Rheumatoid arthritis	39 (43.8)	6 (54.5)	33 (42.9)
Spondyloarthropathy	5 (5.6)	1 (9.1)	4 (5.1)
First language			
English	57 (86.3)	4 (50)	53 (91.4)
Other	9 (13.7)	4 (50)	5 (8.6)
Ethnicity			
White British	55 (61.1)	4 (33.3)	41 (65.3)
Other ethnic groups	35 (38.9)	8 (66.7)	27 (34.7)
- Indian	17 (18.9)	5 (41.7)	12 (15.4)
- Bangladeshi	4 (4.4)	2 (16.7)	2 (2.6)
- Pakistani	4 (4.4)	-	4 (5.1)
- Black Caribbean	3 (3.3)	-	3 (3.8)
- White other	3 (3.3)	-	3 (3.8)
- Other mixed backgrounds	2 (2.2)	1 (8.3)	1 (1.3)
- Asian other	1 (1.1)	-	1 (1.3)
- Mixed: White and the Black Caribbean	1 (1.1)	-	1 (1.3)

Ethnic minority patients were more likely to reject biologics

**Figure 1:**
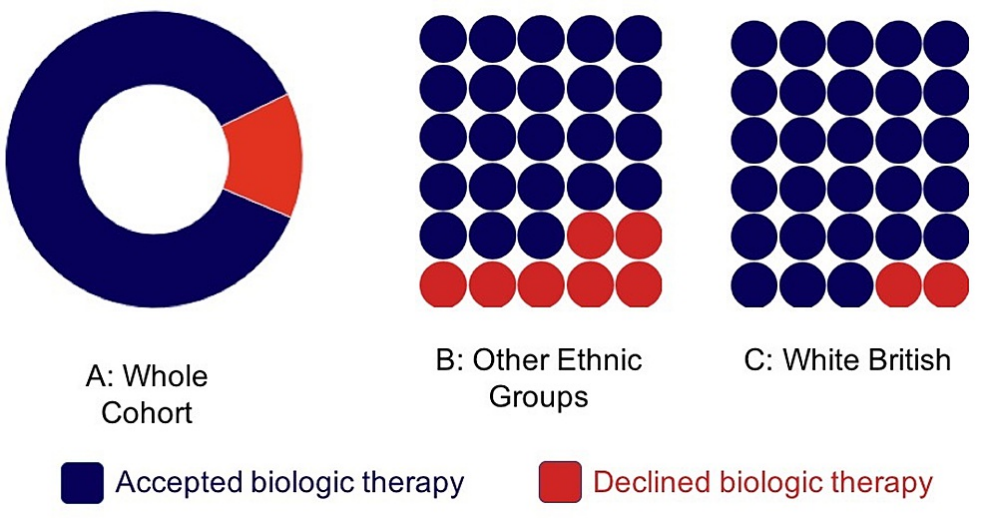
Proportion of patients who declined biological therapies (A) 13.6% of the whole cohort decline biologic disease-modifying drugs when it was offered as standard of care; (B) one in four patients from ethnic groups other than White British declined biologics when offered; (C) seven out of 100 White British patients declined biologics when offered.

Totally 13.1% of the whole cohort rejected biologics (79 accepted, 12 rejected). Both groups (those who accepted and rejected a biologic agent) were relatively well matched for age, sex, and diagnosis (Table 1). The cohort who rejected a biologic agent was over-represented by patients from OEG (66.7% vs 34% among those who accepted treatment) and those who did not speak English as a first language (50% vs 8.6%). The rejection rate among the White British (WB) population was 7%, increasing to 25% among OEG (Figure 1).

In keeping with this, an unadjusted binomial regression model confirmed a strong association between the likelihood to accept treatment with OEGs (OR 0.265, CI 0.73-0.959, p = 0.043) and those who did not speak English as a first language (OR 0.094, CI 0.18-0.497, p = 0.005). Both reduced the odds of a patient accepting biological disease-modifying therapy being offered as a standard of care. As the demographic data collected were well matched between the two groups, no adjustments were made to the model on this basis. First language spoken also correlated strongly with ethnicity (R2 = 0.3, and R2 = 0.8, P < 0.001), and was therefore unlikely to be an independent variable. As such was not included as an adjustment in the model.

## Discussion

Rheumatological disease outcomes vary with ethnicity [10,11] and addressing this is crucial for equitable care. While variations may be partially explained by biological factors such as single-nucleotide polymorphisms [3], engagement with treatment is likely a major contributor [12].

Much of the literature with regards to medication engagement focuses on adherence. Although useful, adherence is assessed through self-reporting, or proxies such as prescription refills and pill counts [9] which are potentially inaccurate. Uptake is a more convenient measure and is by nature less prone to subjectivity being binary in the outcome. We believe that if discrepancies in the uptake of biologics exist between ethnicities, in a healthcare system free at the point of access, it is likely to allude to cultural, economic, and language barriers that may have negative impacts on adherence and disease outcomes.

Our results demonstrated a disparity between White British and other ethnicities in the uptake of biologics. Explanations for this discrepancy have historically been ascribed to factors such as education or socioeconomic status in the context of adherence [2,13,14]. However, Lee et al. found ethnic minorities in North America were less likely to be on biologics even after adjusting for these confounders [1]. Our observations here are thus likely to have complex, multifactorial explanations. Kumar et al. for instance, found a discordance in the perceived longevity of RA between South-Asian patients and clinicians, with South-Asians viewing RA as a short-term ailment [2].

Of significance, we noted that all OEG patients rejecting biologics did not speak English as their first language. Our results are however caveated by first language only being documented in 70 out of our 91 cases. While this observation does not necessarily mean that communication was hindered, it hints at the complex interplay of cultural factors that may influence decision-making. This finding and interpretation are buttressed by similar reports in the literature with regards to DMARD adherence [2,13]. Although understanding the relationship between the first language spoken and the acceptance of biologic therapy was outside the remit of this study, it is nevertheless hypothesis-generating, and qualitative studies to investigate this further are encouraged.

Significant challenges can thus be encountered when counselling patients from different ethnic backgrounds. While this process routinely does and should include interpreters, motivational interviewing, leaflets, and audio-visual aids where appropriate [15,16], effective counselling may also require healthcare workers to gain deeper insights into different cultures that may be pertinent to their locality. This may encourage considerate dialogue, indeed patients who feel their beliefs are overlooked are more likely to reject therapy [17].

A patient’s locus of control is also an important consideration [18]. The impact of ‘decision makers’ in families can vary significantly by culture, but can heavily influence the decision to accept therapy. Anecdotally, we find this to be a particular challenge among cultures where traditional medicine has strong roots. It may thus be prudent to accommodate family decision-makers during counselling, if appropriate.

Elucidating causes for the disparities we observed was outside the scope of our study and requires qualitative research. Given the subjectivity and significance of this subject, however, we encourage all centres to investigate local disparities in the uptake of evidence-based treatments.

Our study was mainly limited by its sample size. This prevented more detailed analyses from being performed, including relationships with socioeconomic status which could have been inferred from postcodes from which England’s index of multiple deprivations could have been derived. Expanding our numbers could allow for this to be corrected in an adjusted regression model. Understanding the precise reasons for why therapy was declined was also beyond the scope of this study, and warrants further investigation through focus groups or semi-structured interviews. This will allow for factors influencing treatment acceptance among different ethnic groups to be clearly defined and addressed.

## Conclusions

In conclusion, we demonstrate a disparity in the acceptance of biologic DMARD between the White British population and patients from other ethnicities. The reasons for this are likely to be multifactorial and will need further evaluation to be addressed effectively. Tackling this issue is imperative to reducing preventable disparities in the outcomes of rheumatological disease between different ethnicities.
